# Parkinson’s Disease and Bipolar Disorder: a case report and narrative review

**DOI:** 10.1192/j.eurpsy.2022.1219

**Published:** 2022-09-01

**Authors:** H. Andreu Gracia, L. Ilzarbe, O. Marco Estrada, L. Bueno Sanya, O. De Juan Viladegut, L. Olivier Mayorga, L. Pintor, F. Valldeoriola, I. Grande

**Affiliations:** 1 Hospital Clínic de Barcelona, Psychiatry, Barcelona, Spain; 2 Hospital Clínic de Barcelona, Consultation Liaison Psychiatry, Barcelona, Spain; 3 Hospital Clínic de Barcelona, Neurology, Barcelona, Spain

**Keywords:** bipolar disorder, differential diagnosis, comorbidity, Parkinson’s Disease

## Abstract

**Introduction:**

Bipolar disorder (BD) is considered a risk factor for developing Parkinson’s Disease (PD) because of an altered dopamine activity in both entities. Comorbidity may delay diagnosis and difficult therapeutic management.

**Objectives:**

To describe the case of a patient with both BD and PD and to determine the appropriate diagnostic and therapeutic approach for patients presenting both entities.

**Methods:**

We present the case of a 58-year-old woman attended in our neurology unit due to the initial presence of visual hallucinations as a core symptom.

**Results:**

Psychotic symptoms as hallucinations and off-times, frequently observed in PD, may be misdiagnosed with a worsening of depressive polarity of BD. Thus, overlap between symptoms may lead to a challenging differential diagnosis. Moreover, there is no consensus about the therapeutic management of the comorbidity, due to the bidirectional worsening of symptoms when treatment is adjusted. In our case, a diagnosis of dopaminergic psychosis was made so antipsychotic treatment with quetiapine 50 mg/d was initiated. A worsening of symptoms was observed, presenting the patient a stuporous status, mutism and generalized rigidity. Neuroimaging and lumbar puncture were performed showing no alterations; electroencephalogram showed diffuse slowing. Final diagnosis was an off-episode of PD and a multifactorial encephalopathy resulting in visual hallucinations.

**Conclusions:**

Coexistence of PD and BD may lead to a diagnostic and therapeutic delay and therefore a worse prognosis. Although these diseases are well-known, it is still challenging to manage patients presenting both entities. Further research is needed to clarify the proper diagnostic and therapeutic approach for these patients.

**Disclosure:**

No significant relationships.

